# *C. albicans* pseudouridine glycosidase is important for growth in hygromycin stress and for filamentation

**DOI:** 10.17912/micropub.biology.001713

**Published:** 2025-08-21

**Authors:** Rheanna E. Walther, Emeline Singh, Douglas A. Bernstein

**Affiliations:** 1 Biology, Ball State University, Muncie, Indiana, United States

## Abstract

When an RNA is no longer needed or has become damaged, it is degraded to its single base components. Pseudouridine is found in all domains of life and is found in a variety of types of RNA. Pseudouridine has ribose and uracil moieties attached via a C-C bond. Cleavage of this bond is performed by pseudouridine glycosidases. We find deletion of the pseudouridine glycosidase from the human fungal pathogen *C. albicans* leads to sensitivity to hygromycin and changes to filamentation. In addition, deletion of pseudouridine glycosidase leads to the upregulation of several permeases. Our data suggest pseudouridine glycosidases are important for *C. albicans* fitness.

**Figure 1. Phenotypic assessment of PUG1 , TNA12 , and GAP2 mutants:**
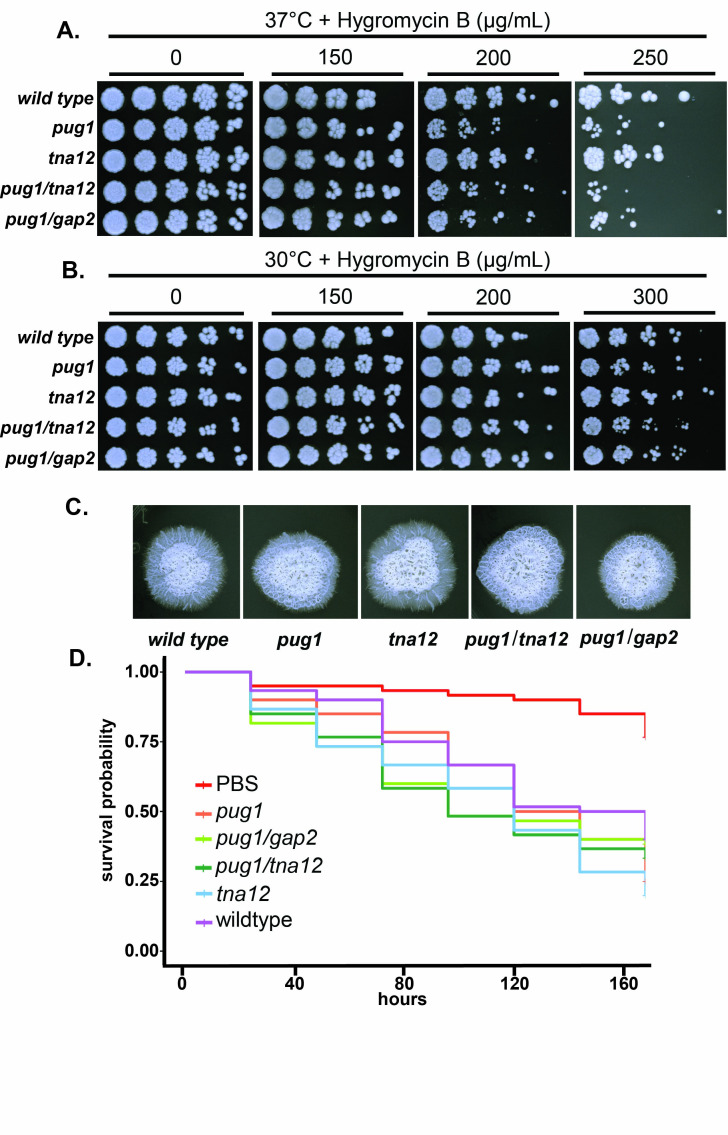
A. A four-fold dilution series on Yeast Peptone Dextrose (YPD) was plated using a 48-pin replicator onto YPD supplemented with 0, 150, 200, 250, or 300 µg/mL Hygromycin B. YPD-only plates were incubated for two days and YPD with Hygromycin B were incubated for three days at 37°C prior to imaging B. same as A. but 30°C. C. Single C. albicans cells were isolated to a quadrant of a petri dish of Spider media. The plates were imaged after ten days of growth at 37°C. D. Infection of Galleria mellonella larvae. Ten thousand C. albicans cells suspended in sterile PBS, or sterile PBS alone was injected into the left proleg. n = 60 larvae were infected per treatment group divided evenly over six biological replicates. Survival was assessed daily and recorded on a Kaplan-Meier survival plot.

## Description

Pseudouridine, the most prevalent RNA modification, is found in all domains of life (Spenkuch, Motorin, & Helm, 2014). Pseudouridine is formed by pseudouridine synthases that cleave the carbon nitrogen glycosidic bond of uridine reattaching the uracil moiety at the C1 position forming a carbon-carbon glycosidic bond (Rintala-Dempsey & Kothe, 2017). This rearrangement exposes nitrogen allowing it to become an extra potential hydrogen bonding partner. This rearrangement allows pseudouridine to take on conformations distinct from those formed by uridine and thus allows it play important roles in a variety of biochemical processes including translation (Tomikawa, 2025; Zhao, Rai, & Li, 2023). Pseudouridine is found in rRNA, tRNA, mRNA and snRNAs(Carlile et al., 2014; Lovejoy, Riordan, & Brown, 2014; Schwartz et al., 2014) and recently, pseudouridine and its modified analogs have been shown to be important for the stabilization of mRNA vaccines(Morais, Adachi, & Yu, 2021).

When an RNA is no longer needed or has become damaged, it is degraded to its single base components (Dowdle & Lykke-Andersen, 2025). After this, the C-N glycosidic bond is cleaved, and bases and ribose are funneled into other metabolic processes. Pseudouridine poses a challenge for these enzymes, as it contains a C-C glycosidic bond and as such is largely resistant to cleavage. In some organisms, such as mammals pseudouridine can be excreted (Tamura et al., 1988). However, in other organisms pseudouridine glycosidase enzymes degrade pseudouridine. Pseudouridine glycosidase enzymes are found in many plants(Lee, Kim, & Rhee, 2024), fungi, archaea, and eubacteria (Thapa, Oja, & Metsa-Ketela, 2014). Enzymatic mechanisms for these enzymes have been described (S. Huang, N. Mahanta, T. P. Begley, & S. E. Ealick, 2012), but little is known about the biological role these families of enzymes play *in vivo* . Pseudouridine glycosidases act in coordination with a pseudouridine kinase, and it has been shown that these enzymatic activities can be either linked on one multifunctional enzyme, or present on distinct polypeptides (Chen & Witte, 2020). As this enzymatic activity is not found in mammals, it is possible that inhibition or manipulation of this activity could be leveraged as a therapeutic.

Here we report the first characterization of a budding yeast pseudouridine glycosidase, *C. albicans PUG1* . We find that knockout of *PUG1* does not have deleterious effects on growth under permissive conditions, but we do find knockout can lead to growth defects during translation stress. Further, we determined that only two transcripts were found in higher quantities in the *PUG1* knockout. Double knockouts of these two genes with *PUG1* did not have a significant effect on phenotype. Furthermore, we found that *PUG1* knockout did not have a significant effect on *C. albicans* virulence in a wax moth model. Together, our results suggest that pseudouridine degradation does not play a critical role in *C. albicans* fitness in the permissive environments.

Pseudouridine glycosidase and kinase activities have been shown to be harbored on either a single protein or two distinct polypeptides with each having a either kinase or glycosidase activity(Chen & Witte, 2020; Thapa et al., 2014). Examination of the *C. albicans* genome suggested that orf 19.6187 is an open reading frame of 2026 residues encoding a bifunctional enzyme harboring both pseudouridine glycosidase and pseudouridine kinase domains separated by an intron of 52 bases. This arrangement is supported by the presence of 5’ and 3’ splice recognition sites and the conservation of this arrangement in other closely related *Candida* species. Interestingly, more distantly related species such as *C. lusitaniae* and *C.* *guilliermondii* appear to lack the C-terminal kinase domain(Lew-Smith, Binkley, & Sherlock, 2025; Skrzypek et al., 2017).

We used CRISPR mediated genome editing to insert stop codons in the 5’ end of the gene generating a functional knockout, *pug1* (Vyas et al., 2018). After making these functional knockouts of *PUG1* we tested if elimination had any effect on *C. albicans* fitness. We grew *C. albicans* on YPD at 30 and 37°C and found that the mutants grew just as well as the wild type strain Figure 1 A and B. Since pseudouridine is important for a variety of aspects of translation we next decided to test if the mutant strain was sensitive to translational stress by growing them in the presence of hygromycin, a translational inhibitor(Borovinskaya, Shoji, Fredrick, & Cate, 2008; Cabanas, Vazquez, & Modolell, 1978). We found that *pug1* was sensitive to hygromycin mediated translational stress. This sensitivity for more pronounced at 37 than at 30°C Figure 1 A and B.

Next, we identified genes that were up or down regulated in the *pug1* mutant. We found that only a modest number of genes were differentially regulated in *pug1* at 37°C STable 2. We did not include hygromycin in these cultures as hygromycin has a profound effect on metabolism would have made it difficult to identify genes that were differentially regulated because of the mutation. Interestingly we found that only two genes were significantly upregulated in the mutant, *TNA12* and *GAP2* STable2. *TNA12* while conserved in fungi has no mammalian homolog and is thought to transport carboxylic acids out of the cell (Llorente & Dujon, 2000). *GAP2* is a broadly conserved amino acid permease with homologs in both other fungi and mammals (Kraidlova et al., 2016). We proceeded to generate strains *tna12/pug1* , *tna12* , and *gap2/pug1* . We screened hundreds of transformants in an attempt to isolate a *gap2* single mutant but were unsuccessful. We found *tna12* knockout does not have a profound effect on fitness even in the presence of hygromycin and both double knockouts of *pug1* and either *tna12* or *gap2* did not significantly exacerbate the fitness defect observed in the *pug1* single knockout Figure 1 A and B.

We next tested if our mutants influenced filamentation, a key virulence trait. We grew our cells on Spider agar, a medium known to induce *C. albicans* filamentation (Liu, Kohler, & Fink, 1994). We found that *pug1* had particular aspects of filamentation that were depressed, such as agar invasion, in comparison to wild type Figure 1 C. However, knockout did not completely abrogate filamentation as the colonies maintained a wrinkled phenotype Figure 1 C. Double knockouts *tna12/pug1* or *gap2/pug1* displayed similar phenotypes to *pug1* maintaining the wrinkled colony phenotype while demonstrating less agar invasion than wild type. *tna12* mutants displayed wild type filamentation under these conditions. We next tested if filamentation was inhibited in liquid media but did not observe defects in either Spider media or RPMI serum at 37 or 30°C 2, 4, or 6 hours. As we observed decreases in growth under stress and filamentation we next wanted to test if *pug1* had a defect in virulence in a *Galleria mellonella* wax moth model. We found that *pug1* , *tna12/pug1* , *tna12* , and *gap2/pug1* virulence were not significantly different from wild type *C. albicans* in the wax moth model Figure 1 D.

Inspection of the *C. albicans* genome suggests *C. albicans* pseudouridine glycosidase and kinase activities are found on a single protein and are important for *C. albicans* growth on hygromycin Figure 1 A and B. Furthermore, certain aspects of filamentation such as agar invasion are adversely affected in the *pug1* mutants but in our wax moth virulence model all our mutant strains *pug1* , *tna12/pug1* , *tna12* , and *gap2/pug1* are equally virulent to wild type Figure 1 C and D. Taken together, this data suggests that *C. albicans* pseudouridine glycosidase plays an important role in some aspects of *C. albicans* biology but under permissive growth conditions is not likely essential.

Degradation of pseudouridine is thought to require both kinase and glycosidase activity (Siyu Huang, Nilkamal Mahanta, Tadhg P. Begley, & Steven E. Ealick, 2012). In bacteria these activities are located on two peptides while in some eukaryotes these activities are harbored on a single protein (Chen & Witte, 2020). Examination of the sequence in *C. albicans* suggests the latter with the two protein encoding domains separated by an intron. While there is a potential additional start codon at the beginning of the 3’ exon we don’t believe that two distinct proteins are likely translated. First, other closely related *Candida* species also appear to encode introns that separate these domains. Interestingly it appears the kinase, which is encoded by the 3’ exon is less conserved in distant *Candida* species. This could mean that kinase function is no longer necessary in these species, or that this function is complemented by another kinase located elsewhere in the genome.

*TNA12* and *GAP2* are found in greater abundance in the *pug1* mutant STable 2, and both proteins are predicted to play roles in transport of biomolecules through the cell membrane. *TNA12* is a putative carboxylic acid transporter while *GAP2* is a putative amino acid permease, and both are highly conserved proteins in the *Candida* fungal clade. One possible model that explains these results is that these transporters are necessary for exporting excess pseudouridine out of the cell in the absence of Pug1. We predict that in *pug1* under stress, pseudouridine will accumulate in the cytoplasm as RNA is degraded. In wild type *C. albicans,* this pseudouridine is degraded by Pug1. In the mutant, pseudouridine cannot be degraded and must be removed from the cytoplasm, so it does not interfere with cellular processes. Up regulation of transporters is one possible mechanism through which this could occur. We found several genes were modestly down regulated in *pug1* . However, the modest down regulation and lack of conserved themes among these genes made it difficult to develop models of how down regulation of these genes would counterbalance the absence of Pug1 activity. As such, we did not pursue further genetic or biochemical interrogation of these genes.

The absence of pseudouridine glycosidase activity from mammals and their presence in nearly all major fungal and bacterial pathogens make them an intriguing potential antimicrobial drug target. Our data suggests that *C. albicans* pseudouridine glycosidase plays important roles in metabolism when *C. albicans* is under translational stress. However, under permissive conditions elimination of this enzyme activity is not detrimental to growth. While this data suggests pseudouridine degradation may not be a suitable drug target for *C. albicans* , it is possible that under the conditions found in a mammalian host pseudouridine degradation is essential and could be a viable therapeutic target. Furthermore, our data does not eliminate the possibility that pseudouridine degradation could be a suitable drug target in other fungal or bacterial pathogens. Nearly all bacterial and fungal pathogens encode a pseudouridine glycosidase and while these enzymes have not yet been shown to be essential, only a few have been investigated. Further examination of this enzyme family will be necessary to determine if these enzymes could provide a novel target for antimicrobial therapeutics.

## Methods

Strain Generation and Validation

All mutant strains used were made using CRISPR-Cas9 mediated genome editing developed by Vyas et al 2018. Both *pug1* and *tna12* used SC5314 (wild type) as the parent strain. Both *pug1tna12* and *pug1gap2* used *pug1* as the parent strain. gRNA sequences (STable 1) were cloned into pV1393 for *TNA12* and pV1524 for *PUG1* and *GAP2* Vyas et al 2018. A 100 bp repair template (STable 1) was cotransformed to remove the PAM site and replace it with a stop codon and PacI restriction site. Digestion with PacI was used to validate stop codon insertion during colony screening. Correct insertion was confirmed by Sanger sequencing. Cas9 guide RNA cassette was removed by activation of flippase and subsequent recombination at flanking FRT sites as described in Vyas et al 2018.

Growth Assays

Wild type and mutant strains were grown in liquid YPD with 0.27mM uridine overnight at 30°C. Cultures were diluted to OD _600_ of 0.1 and 4-fold serial dilutions were plated using a 48-pin replicator on solid YPD media supplemented with Hygromycin B at 37°C. Growth was assessed and imaged daily.

Filamentation Assays

Cells were grown in the same manner as they were in the growth assays except hygromycin was not added to the media and cells were plated on Spider media.

Transcriptomics Analysis

Three biological replicates of SC5314 and *pug1/pug1* RNA were extracted and purified using acid phenol. 5 mg total RNA was polyA-purified and sequenced by Genewiz. The sequencing configuration was HiSeq 2x150bp. Reference genome SC5314 version A22-s07-m01-r20 was used for analysis. Analysis was performed by Genewiz using their standard bioinformatics workflow to identify significantly differentially expressed genes.

Virulence assays

*Galleria mellonella* larvae were supplied by Speedyworm.com. Healthy larvae weighing between 250 and 300 grams were selected. *C. albicans* cultures were grown to early log phase at 37°C. 1x10 ^7^ cells were washed and resuspended in 1 mL of sterile PBS. Each larva was injected with 10 µL of the cell suspension, n=10 per treatment group. Larvae were then housed in sterile glass petri dishes at 37°C. Survival was recorded every 24 hours on a Kaplan-Meier survival curve and was analyzed in R studio using the Log-rank test. This was repeated for a total of six biological replicates.

## Data Availability

Description: Primers used in this study.. Resource Type: Text. DOI: https://doi.org/10.22002/55r7g-fv575 Description: Significant transcript changes . Resource Type: Dataset. DOI: https://doi.org/10.22002/v4meh-gwc40 Description: All transcript chanegs . Resource Type: Dataset. DOI: https://doi.org/10.22002/e247a-ke937 Description: Volcano plot illustrating significant changes. . Resource Type: Image. DOI: https://doi.org/10.22002/0217m-7z533
